# Disseminated intravascular coagulation as a complication after transvenous lead extraction for defibrillator-associated endocarditis: A case report

**DOI:** 10.1016/j.hrcr.2022.02.001

**Published:** 2022-02-10

**Authors:** Heather Dunn, Blair W. Foreman

**Affiliations:** ∗ The University of Iowa College of Nursing, Iowa City, Iowa; † Cardiovascular Medicine, PLLC, Davenport, Iowa

**Keywords:** Cardiac implanted electronic device extraction, Cardiac implanted electronic device system infection, Disseminated intravascular coagulation, Endocarditis, Laser lead extraction

## Introduction

As the number of cardiac implanted electronic devices (CIED) has dramatically increased in the United States,^1^ so has the frequency with which CIEDs are extracted.^2^ Of the 1.2 to 1.4 million CIED devices that are implanted annually worldwide,^1^ 1%–2% of these will require system extraction.^3^ Indications for extraction of CIEDs are well established, with system infection identified as mandatory.^1^^,^^4^ Major intraprocedural complications associated with CIED extraction are well documented.^5^ The risk factors associated with intraprocedural complications related to CIED extraction remain elusive and conflicting, with advanced lead age and female sex consistently identified in the literature as predictors of major complications.^5^^,^^6^ While Sood and colleagues^4^ report that infection was a predictor of major intraprocedural complications, predictors of postprocedural complications remain limited. Disseminated intravascular coagulation (DIC) has been identified as a very rare procedural-related complication related to CIED extraction, but the specifics of clinical presentation and outcomes are unknown.^6^ The 2017 expert consensus statement from the Heart Rhythm Society indicates that complications are defined not only by severity of the complication, but also by timing in relation to the procedure.^1^ Therefore, we report a case of a 78-year-old White male patient with a biventricular implantable cardioverter-defibrillator (ICD) with active and abandoned leads, presenting with *Streptococcus agalactiae bacteremia* necessitating system extraction, culminating in postprocedural DIC.

**Key Teaching Points**

• 1%-2% of cardiac implanted electronic devices (CEID) worldwide will require system extraction, with system infection a mandatory indicator for removal.
• Disseminated intravascular coagulation is a very rare post-procedural complication of CEID extraction.
• Patients undergoing CEID extraction secondary to bacteremia with associated system infection are at risk for sepsis-associated hypofibrinolytic type DIC or surgery-associated consumption DIC with profound bleeding.
• DIC should be considered early if excessive, uncontrollable post-procedural bleeding occurs after CEID system extraction.
• In patients with postoperative hemostasis concerns and difficult to control bleeding, clinicians should intervene quickly to evaluate global coagulation function including hemoglobin and hematocrit, platelet count, prothrombin time, and partial thromboplastin time, but a fibrinogen level and fibrin related markers.

## Case report

### Patient description and case history

**Figure 1:**
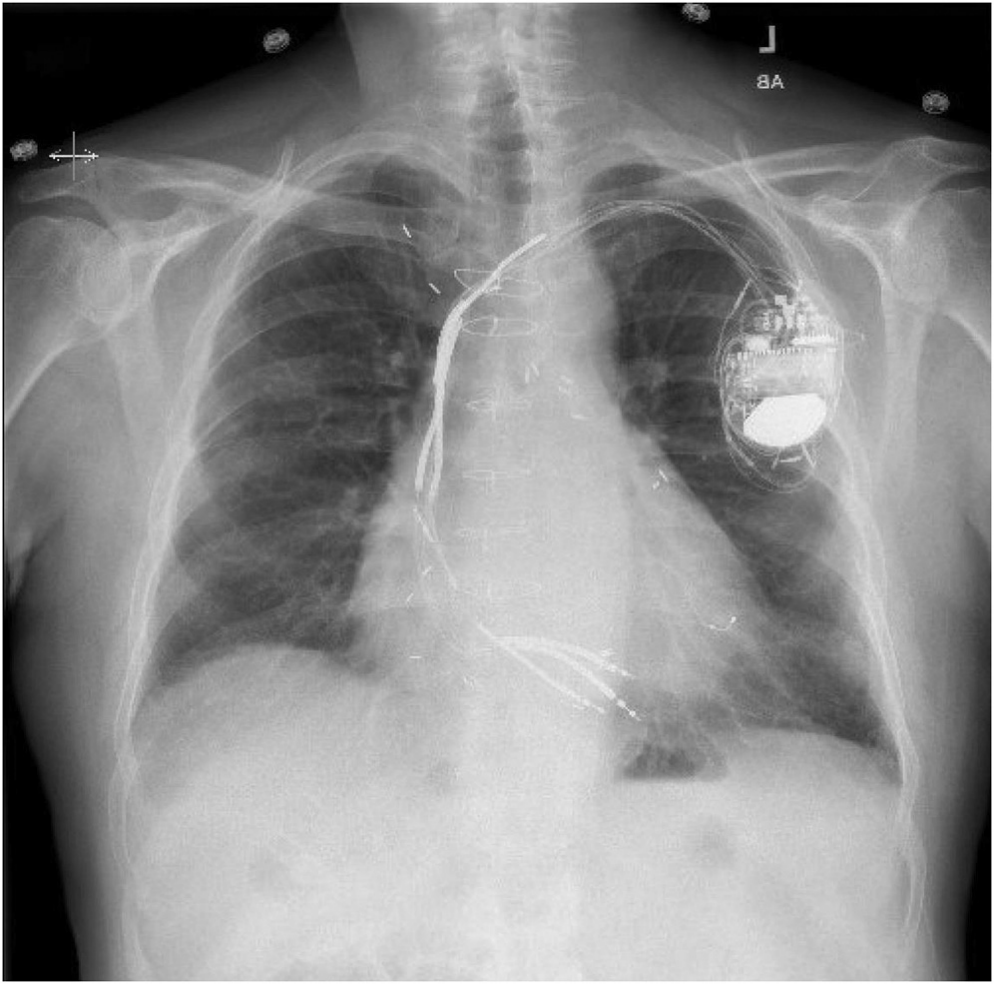
Preprocedural chest radiograph of biventricular implantable cardioverter-defibrillator with active and abandoned leads.

The patient was a 78-year old White man with a past medical history of sudden cardiac death with atherosclerotic coronary vascular disease, followed by coronary artery bypass grafting in 1999, peripheral vascular disease, hyperlipidemia, hypertension, transient ischemic attack, heart failure with reduced ejection fraction (most recent ejection fraction prior to admission 15%), and ICD implanted in 2005 for secondary prevention, upgraded to a biventricular ICD in 2012, with abandoned leads secondary to fracture (Figure 1). He presented to an outside hospital with complaints of rigors, sweating, and temperature of 104°F. Leukocytosis of 13,000/μL was noted, and the patient was initiated on intravenous vancomycin and meropenem and admitted for sepsis. Initial blood cultures were 2 out of 2 positive for *Streptococcus agalactiae*, antibiotics were de-escalated to vancomycin only, and the patient underwent transesophageal echocardiography, which demonstrated vegetation on the pacemaker leads in the right atrium. Seventy-two hours after admission, the patient was transferred to our facility for consultation with an experienced electrophysiologic extractionist.

### Physical examination upon presentation

Upon transfer, the patient was without acute distress. His right lower extremity was mildly edematous and erythematous from knee to ankle, consistent with cellulitis and likely source of infection. His ICD site was without swelling or erythema. Laboratory results are detailed in Table 1. Repeat blood cultures obtained on transfer were without growth. Vital signs were within normal range, and without fever. Home medications included dual antiplatelet therapy and a direct oral anticoagulant, which were discontinued upon hospitalization. Therapeutic-dose low-molecular-weight heparin was initiated and maintained until 18 hours prior to extraction. Interrogation of the ICD revealed a functional biventricular device with a bipolar coronary sinus lead and 2 abandoned leads. The patient was continued on intravenous vancomycin while awaiting multidisciplinary coordination for CIED extraction.

**Table 1:** Laboratory results

Lab test	Admission	Preop	Rapid response (10:38 PM)	ICU admission (2:49 AM)	ICU AM day #1 (8:45 AM)	Discharge
Na^+^, mmol/L	141	139	142	157	143	135
K^+^, mmol/L	4.0	4.1	4.2	4.1	3.7	4.2
Cl^-^, mmol/L	108	103	107	110	105	101
CO_2_, mmol/L	23	28	23	39	22	26
BUN, mg/dL	10	10	9	13	14	14
CRT, mg/dL	0.80	0.86	0.88	1.16	1.26	0.68
Ca^+^, mg/dL	8.9	8.7	7.9	7.6	8.2	8.7
ALT, IU/L	45					
AST, IU/L	40					
ALP, units/L	57					
Lactic acid				8.1	9.0	
Transferrin				129		
WBC, thousands/μL	6.77	4.8	22.52	29.04	18.46	7.19
Hgb, g/dL / HCT	12.1 / 36.4%	11.7 / 35.9%	9.7 / 30.6%	9.2 / 28.1%	5.8 / 17.5%	10.8 / 33.3%
Platelets, thousands/μL	105	227	157	85	82	220
PT, seconds	10.9	11.6		10.1	14.3	10.7
INR, IU	1.0	1.1		0.9	1.4	1.0
PTT, seconds	30			41	33	
Fibrinogen mg/dL				<70	198	402
D-dimer, m/L				>32.5		
Arterial blood gases						
pH				7.40		
PCO_2_				40.7		
PO_2_				76		
HCO3^-^				25.1		

ALP = alkaline phosphatase; ALT = alanine aminotransferase; AST = aspartate aminotransferase; BUN = blood urea nitrogen; CRT = creatinine; HCT = hematocrit; Hgb = hemaglobin; ICU = intensive care unit; INR = international normalized ratio; Preop = preoperative; PT = prothrombin time test; PTT = partial thromboplastin time test; WBC = white blood cell.

### Extraction

**Figure 2:**
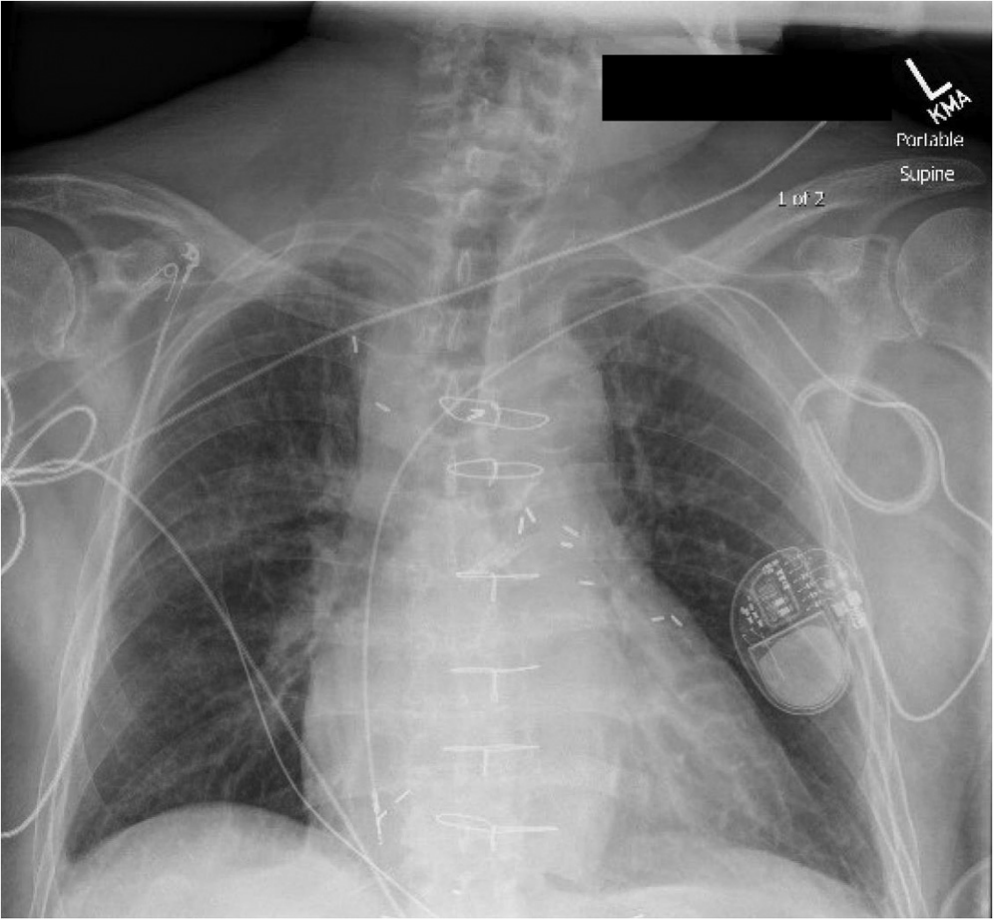
Postprocedural chest radiograph revealing removal of all biventricular implantable cardioverter-defibrillator leads, insertion of temporary wire to the right ventricular apex, and noncentral peripherally inserted vascular access device.

Seven days after transfer, the patient was taken to the operating room for 5-lead extraction of the ICD system and debulking of the defibrillator pocket under general anesthesia. Ultrasound-guided vascular access included right femoral arterial line for blood pressure management; right femoral vein 6F venous access for temporary right ventricular pacing; left femoral vein 9F venous sheath for intracardiac echocardiogram of the right atrium; and a right femoral 12F venous sheath through which an occlusive bridging balloon was delivered over wire to the superior vena cava. The left deltopectoral pocket was debulked via blunt and sharp dissection with electrocautery for hemostasis. All leads were freed and removed via standard mechanical traction and laser lead assistance via expandable Spectranetics® laser lead stylets (Koninklijke Philips, N.V.). A temporary wire was placed to the right ventricular apex, externalized, and connected to a pacemaker in a temporary permanent configuration (Figure 2). Hemostasis was confirmed. The left deltopectoral pocket was irrigated, a Jackson-Pratt drain was placed, and standard wound closure was performed. All venous and arterial sheaths were removed. Procedure duration was 4.5 hours in total length. The patient was extubated per anesthesia and transported to the postanesthesia recovery unit in stable condition at 7:56 PM. Upon transfer to the cardiac unit at 9:15 PM his vitals were as follows: pulse 64 beats/min, blood pressure 113/59, 100% SpO_2_ on room air.

### Postprocedural events

Two and a half hours postprocedure, the extracting electrophysiologist was notified that the Jackson-Pratt surgical drain was draining sanguineous drainage at a rate of 80 mL/h. The patient was suddenly hypotensive with a blood pressure of 60/30. A rapid response was called for persistent hypotension not responsive to fluid challenge and newly developing bilateral groin bleeding. Limited bedside echocardiogram was without evidence of pericardial effusion. Manual pressure held to bilateral groin sites was unable to control the bleeding. Throughout this time, the patient complained of low back spasms and remained alert.

**Figure 3:**
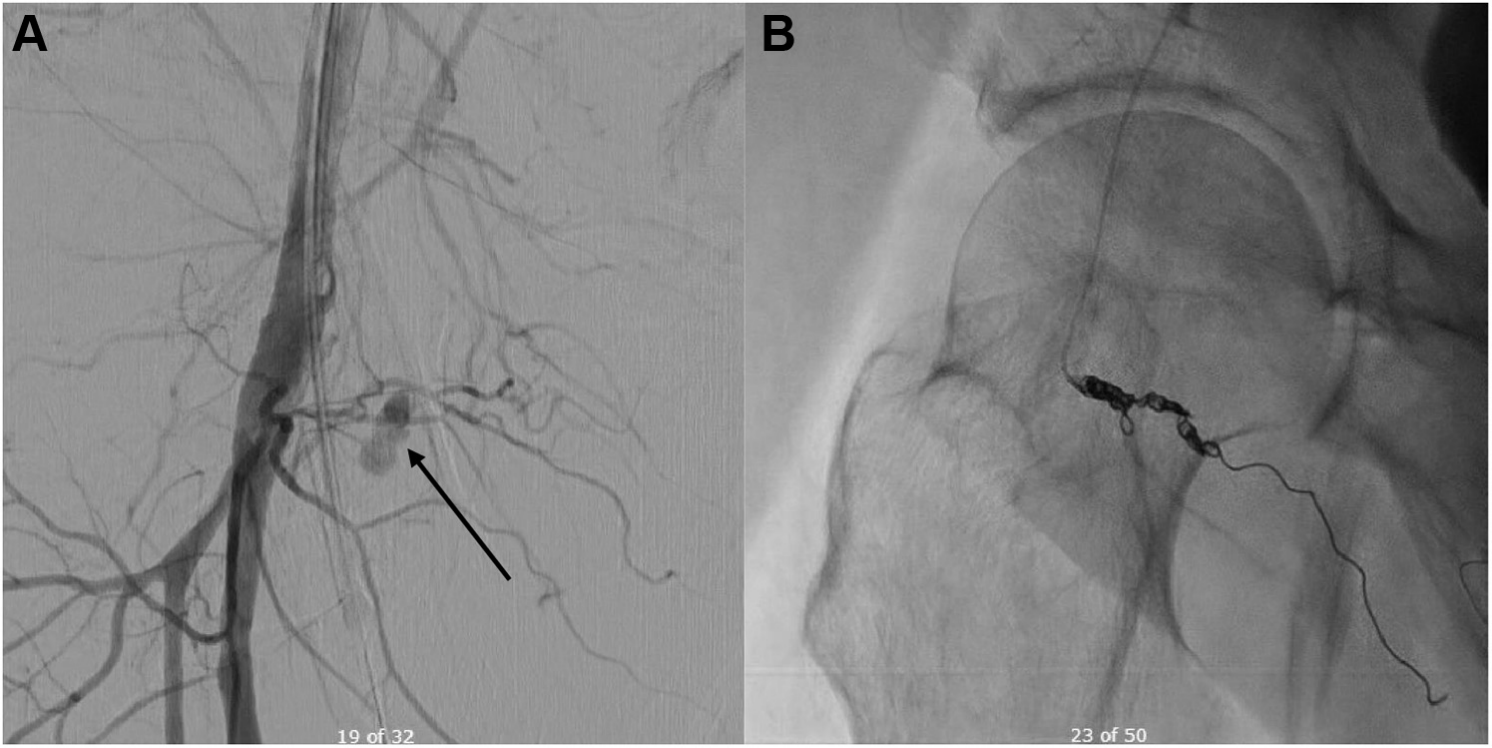
Right common femoral artery angiography indicating persistent bleeding (**A**) and hemostasis post coil embolization (**B**).

Given the inability to control bleeding from the ICD pocket, the patient returned to the operating room for pocket exploration, and a femoral compression device was applied to the right groin while manual compression continued to the left groin. Operative exploration of the old ICD pocket revealed diffuse oozing without an obvious source of bleeding. Additional irrigation and electrocautery for hemostasis was performed. Owing to inability to identify an obvious source of bleeding, the patient was taken from the operating room to radiology for computed tomography of his chest, abdomen, and pelvis. This revealed a large hematoma in the right anterior thigh measuring 4 × 6 × 7 cm with extension into the right groin with evidence of acute extravasation. He remained hypotensive, requiring vasopressor support, and was transferred to the intensive care unit (ICU). Persistent bleeding from multiple sites raised the suspicion of DIC, which was confirmed by laboratory analysis (Table 1) upon admission to the ICU. Replacement therapy with packed red blood cells, fresh frozen plasma, platelets, and cryoprecipitate was initiated. The right groin site continued to bleed uncontrollably, and ultrasound evaluation performed in the morning of postoperative day 1 confirmed a large right groin hematoma with persistent active arterial bleeding. Interventional cardiology was consulted. Angiography of the right common femoral artery (Figure 3) with iliofemoral runoff with coil embolization of the right medial femoral circumflex artery was performed to control the bleeding.

On postoperative day 2, the patient experienced an episode of pulseless ventricular tachycardia requiring cardiopulmonary resuscitation that responded to 1 defibrillation. In response to this episode, he was initiated on intravenous amiodarone but developed secondary bradycardia, necessitating transition from Levophed to dopamine for heart rate support.

### Outcome

Despite a guarded prognosis, the patient continued to improve. His acute bleeding resolved. Fluid volume overload developed but was responsive to diuresis. His intravenous amiodarone was discontinued after 48 hours but he continued to require fixed-dose dopamine for heart rate support until a contralateral ICD with TYRX absorbable antibacterial envelope (Medtronic, Minneapolis, MN) was placed on postoperative day 7. Intravenous antibiotics were managed per the infectious disease service throughout, and the patient received a total of 4 weeks of therapy. Ultimately, he was discharged to a skilled nursing facility for ongoing intravenous antibiotic therapy and physical rehabilitation on postoperative day 10. He was seen in follow-up in the outpatient setting on postoperative day 48, where he reported a full return to his personal residence with his spouse, without limitations on activities of daily living or health-related quality of life.

## Discussion

DIC is defined by the International Society on Thrombosis and Haemostasis as “an acquired syndrome characterized by the intravascular activation of coagulation with loss of localization arising from different causes.”^7^ Recognition and treatment of the underlying cause of DIC is the foundation of DIC treatment guidelines.^8^ Pathologic conditions associated with DIC are well documented in the literature,^7^ but in the context of the present case the most likely triggering events were sepsis combined with surgery. As CIED infection is a primary indication for surgical system extraction,^4^ the combination of these triggering events in relation to the onset of DIC deserves closer attention and evaluation.

In the present case, initial blood cultures at the time of presentation were positive for *Streptococcus agalactiae*, a gram-positive organism known to cause systemic hyperinflammation and hypofibrinolytic-type DIC.^9^ Sepsis-associated DIC is a complex inflammatory response to invading microorganisms that activate intracellular signal-transduction pathways, resulting in the synthesis of proinflammatory cytokines and chemokines, triggering complement activation and the coagulation pathway, resulting in hypofibrinolysis and eventual organ dysfunction.^8^ Circulating inflammatory cytokines promote the production of large amounts of tissue factor from circulating monocytes, macrophages, and the vascular endothelium, leading to marked activation of coagulation.^10^ At the same time, histone-induced vascular endothelial dysfunction occurs during DIC where damaged endothelial cells shift their physiologic anticoagulant properties toward procoagulant tendencies via overexpression of von Willebrand factor, thus inhibiting fibrinolysis during DIC.^11^ Additionally, platelets bind directly via toll-like receptors to *Streptococcus agalactiae* bacteria, resulting in platelet aggregation, adhesion, and upgraded expression of CD62P, culminating in thrombocytopenia,^12^ which was a noted clinical feature in the present case postprocedurally. The imbalance in hypofibrinolytic-type DIC between severe inflammatory-induced coagulation but limited fibrinolytic activation is typical in sepsis and results in multiple microthrombi that culminates in organ dysfunction with relatively mild bleeding complications.^10^ In the present case, the initial postprocedural complication reported by bedside nursing was uncontrolled bleeding, which is atypical for sepsis-associated, hypofibrinolytic-type DIC.

While the patient did develop a right groin hematoma requiring coil embolization hemostasis, this was not the initial site of bleeding reported by bedside nursing. Additionally, laboratory assessment at the onset of the rapid response and ICU admission are without a clinically relevant drop in hemoglobin and hematocrit or metabolic acidosis (Table 1). As such, it is not likely that the right groin hematoma initiated a hemorrhage shock state or the resultant DIC. In contrast, there are known fibrinolytic phenotypes of DIC associated with surgery that are typically associated with a consumption coagulopathy and profound bleeding.^13^ Endothelial damage resulting from surgical intervention exposes tissue factor directly to the circulating blood, resulting in an overwhelming initiation of sustained coagulation, leading to a rapid consumption of coagulation factors, resulting in hemorhage.^14^ Unless associated with profound and sustained hypovolemic shock secondary to uncontrolled blood loss, organ dysfunction is rare.^10^ Consumptive-type DIC associated with major bleeding following surgery^8^ correlates with the clinical postprocedural presentation in the present case.

## Conclusion

Development of DIC following successful CIED extraction has been reported in only 1 published study,^6^ making DIC a very rare postprocedural complication of CIED extraction. To date, details of prior cases are unknown. Early in the process of clinical reasoning and decision-making, a set of diagnostic hypotheses is formulated based on the presenting clinical problem coupled with knowledge of prior cases.^15^ This case is an indication that DIC should be considered early if excessive, uncontrollable postprocedural bleeding occurs after CIED system extraction, prompting early diagnostic evaluation and treatment to prevent complications and mortality. Based on our present case, we recommend that in patients with postoperative hemostasis concerns and difficult-to-control bleeding, clinicians should intervene quickly to evaluate global coagulation function. Laboratory evaluation should include not only a hemoglobin and hematocrit, platelet count, prothrombin time, and partial thromboplastin time, but a fibrinogen level and fibrin-related markers.

## References

[1] Kusumoto F.M., Schoenfeld M.H., Wilkoff B.L. (2017). 2017 HRS expert consensus statement on cardiovascular implantable electronic device lead management and extraction. Heart Rhythm.

[2] Deshmukh A., Patel N., Noseworthy P.A. (2015). Trends in use and adverse outcomes associated with transvenous lead removal in the United States. Circulation.

[3] Wazni O., Wilkoff B.L. (2016). Considerations for cardiac device lead extraction. Nat Rev Cardiol.

[4] Bongiorni M.G., Burri H., Deharo J.C. (2018). 2018 EHRA expert consensus statement on lead extraction: recommendations on definitions, endpoints, research trial design, and data collection requirements for clinical scientific studies and registries: endorsed by APHRS/HRS/LAHRS. Europace.

[5] Sood N., Martin D.T., Lampert R., Curtis J.P., Parzynski C., Clancy J. (2018). Incidence and predictors of perioperative complications with transvenous lead extractions: real-world experience with national cardiovascular data registry. Circ Arrhythm Electrophysiol.

[6] Bongiorni M.G., Kennergren C., Butter C. (2017). The European Lead Extraction ConTRolled (ELECTRa) study: a European Heart Rhythm Association (EHRA) registry of transvenous lead extraction outcomes. Eur Heart J.

[7] Taylor F.B., Toh C.H., Hoots W.K., Wada H., Levi M. (2001). Towards definition, clinical and laboratory criteria, and a scoring system for disseminated intravascular coagulation. Thromb Haemost.

[8] Wada H., Thachil J., Di Nisio M. (2013). Guidance for diagnosis and treatment of DIC from harmonization of the recommendations from three guidelines. J Thromb Haemost.

[9] Siemens N., Oehmcke-Hecht S., Hoßmann J. (2020). Prothrombotic and proinflammatory activities of the β-hemolytic group B streptococcal pigment. J Innate Immun.

[10] Asakura H. (2014). Classifying types of disseminated intravascular coagulation: clinical and animal models. J Intensive Care.

[11] Iba T., Levy J.H. (2020). Sepsis-induced coagulopathy and disseminated intravascular coagulation. Anesthesiology.

[12] Ma L., Xianming L., Li H., Li Y., Shuangfen X., Hui W. (2012). Group B streptococcus induce platelet activation via toll-like receptor 2. Blood.

[13] Blaisdell F.W. (2012). Causes, prevention, and treatment of intravascular coagulation and disseminated intravascular coagulation. J Trauma Acute Care Surg.

[14] Osterud B., Bjørklid E. (2001). The tissue factor pathway in disseminated intravascular coagulation. Semin Thromb Hemost.

[15] Elstein A.S., Shulman L.S., Sprafka S.A. (1978). Medical Problem Solving: An Analysis of Clinical Reasoning.

